# Innovations in the Management of the Difficult Airway: A Narrative Review

**DOI:** 10.7759/cureus.35117

**Published:** 2023-02-17

**Authors:** Binu Ravindran

**Affiliations:** 1 Anaesthesiology, Dartford and Gravesham NHS Trust, Dartford, GBR

**Keywords:** airway training, airway equipment, radiology, diagnostic imaging, difficult airway, intensive care, anaesthesia

## Abstract

The difficult airway (DA) remains a perpetual challenge and its implications have led to multiple advances, technological and otherwise in this area. This article investigates the latest developments in the definition, prediction tools and diagnostics like airway and neck Ultrasonography (USG), Magnetic Resonance Imaging (MRI) and Computed tomography (CT) scans, preoperative Virtual endoscopy (VE) and 3D printing. Innovations in airway devices and adjuncts are analysed. Difficult airway society (DAS) guidelines, American Society of Anaesthesiologists (ASA) Practice Guidelines and Vortex approach for the management of DA are explored. Other breakthroughs include novel oxygen supplementation techniques throughout airway management and tools like Anaesthesia Information Management Systems (AIMS) and Clinical Decision Support (CDS) systems. The delivery of DA training and patient counselling has also undergone vast changes with emerging technology like Virtual Reality (VR), mobile applications and toolkits. The enormous, ever-evolving and endless possibilities in this area have only helped improve clinical standards and enhance patient safety.

## Introduction and background

Unsuccessful airway management is a potentially life-threatening event and one of the most feared situations that anesthetists may encounter. Major airway-related complications occur in one in every 22,000 anaesthetics. Death or brain injury occurs in one in 180,000, with statistical analysis suggesting only 25% of relevant incidents may have been reported [1]. The principal associated adverse outcomes include death, hypoxic brain injury, cardiopulmonary arrest, airway trauma, aspiration of gastric contents, pulmonary oedema and dental damage [1]. The catastrophic complications that ensue with failure of oxygenation or maintenance of a patent airway serve to justify the numerous developments, research, guidelines and algorithms developed in this area. Advances taking place in the delivery of airway training and education reinforce the grave need to learn to deal with this challenge.

The aim of this article was to provide an exhaustive overview encompassing developments in all aspects of DA management. This would include advances in definition, diagnostics, equipment, guidelines, extubation process, training and related technology. Extensive searches were carried out in PubMed and Google Scholar using [‘developments’, ‘devices’, ‘imaging’, ‘guidelines’, ‘training’ or ‘technology’] and [‘difficult airway’ or ‘airway management’] without date limitations. Books, unrelated matters and foreign language material were excluded. The research was also conducted in hard copies of journals, online events, podcast channels and anaesthesia and airway society websites of the UK, USA, Europe, Australia and New Zealand. All information obtained was cross-checked for credibility. Data from airway-related complications were included to highlight the gravity and consequent necessity of developments in this area. Existing literature does not cover all advances in DA management in one paper, nor does it cover evolving aspects in this field. Limitations of this paper are that it is specific to the DA alone and does not include other developments related to airway management in general. Likewise exploring the full extent of practicalities, description, global variation in availability, usage, and pros and cons has not been possible in order to present a compendious review.

## Review

Definitions

According to the 2022 ASA Practice Guidelines for the management of DA, the revised definition of DA is: ‘the clinical situation where a physician trained in anaesthesia care has difficulty or failure in one or more of laryngoscopy, ventilation using a facemask or supraglottic airway (SGA), tracheal intubation, extubation, or invasive airway’ [2]. ‘Difficult intubation is tracheal intubation failing or requiring multiple attempts and difficult laryngoscopy is the inability to visualise any portion of the vocal cords after multiple attempts at laryngoscopy’ [2].

Airway problems could arise due to (a) structural airway deformations, (b) physiological and pathophysiological conditions which risk rapid desaturation and mandate quick securing of the airway, like morbid obesity, pregnancy, conditions with aspiration risk, hypoxemia, hypotension, severe metabolic acidosis and organ failure and (c) miscellaneous factors: circumstances and location, equipment and assistance available, the competence of the clinician and human factors.

Clinical assessment and evaluation

According to the ASA Closed Claims Project report, two-thirds of all claimants with DA were discovered only after induction of anaesthesia [3]. The 4th National Audit Project concluded that failure to assess for and identify potential airway difficulty contributes to a poor outcome [1]. This reinforces the necessity of a thorough preoperative airway assessment comprising a comprehensive history and accurate clinical examination to identify airway problems. DA prediction is enhanced by using a combination of indicators, prediction tools and scoring systems.

Investigations

Clinical assessment alone may fail to accurately predict airway difficulty. Advanced diagnostics refine airway assessment, help formulate precise airway management strategies and guide complex treatment decisions (Table 1). The numerous technological tools are explored below.

**Table 1:** Diagnostic modalities in the prediction of difficult airway (DA)

Modality	Use
X-rays	assess airway structures
USG (Ultrasonography)	identify cricothyroid membrane, DA prediction
CT scan (Computed Tomography scan)	finer characterisation of pathology, image reconstruction
MRI (Magnetic Resonance Imaging)	define soft tissue airway lesions
Nasal endoscopy	bedside visualisation of glottis and subglottic areas
Virtual endoscopy	internal virtual evaluation of airway
3D printing (Three-Dimensional printing)	simulated airway model enables planning, practice and education.

X-rays

X-rays locate and assess airway structures, identify tumours, masses, trauma, foreign bodies, abscesses, epiglottitis, degenerative conditions and tracheal compression or stenosis. Lateral views in neck flexion and extension assess neck movements and detect Atlanto-axial subluxation. They confirm endotracheal tube (ETT) position and help estimate the size and depth of insertion a double-lumen tube (DLT) by measuring the tracheal width digitally using computer software. Various parameters like large anterior osteophytes, ossification of the ligaments, measurements of mandibulohyoid distance, atlanto-occipital distance and extension, mandibular ramus depth and angle, recessed chin and increased tongue mass in radiographs of the head and neck can be used to predict DA [4]. X-rays help rule out or confirm suspicions generated on clinical examination and provide a clearer picture of the location and extent of pathology. They are universally available, easily accessible, cheap and may be the only modality available in resource poor settings. Although not as sensitive and specific as other imaging techniques, X-rays remain a widely used airway imaging modality worldwide.

*Ultrasonography (USG) * 

**Figure 1:**
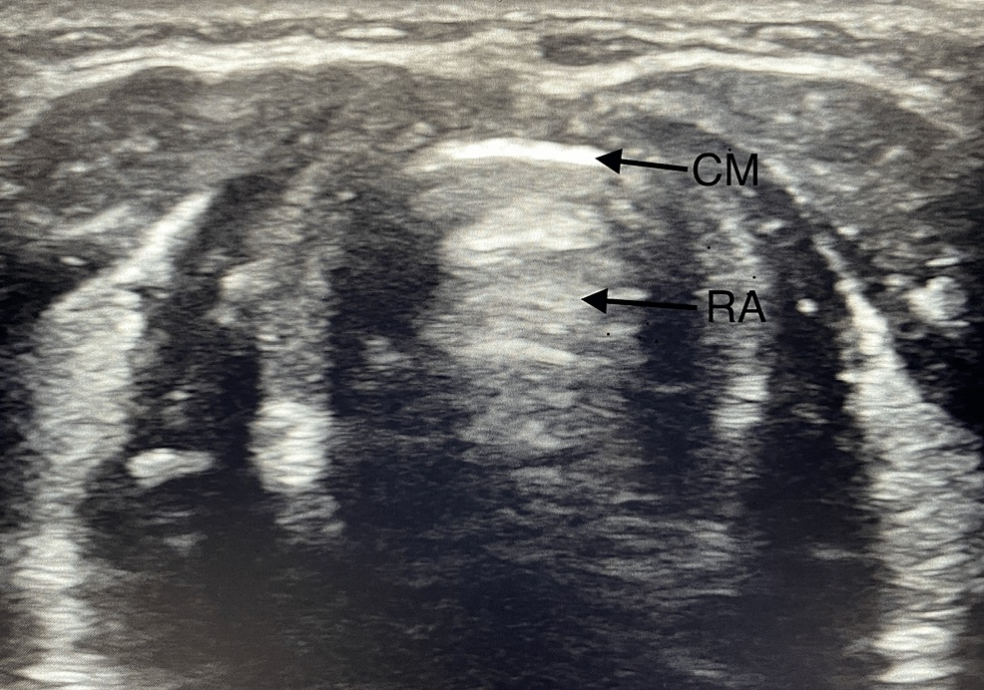
Cricothyroid membrane (CM) in transverse view with reverberation artifacts (RA) at the tissue air border. The image is the original work of the author.

USG is a simple, valuable, portable tool that facilitates non-invasive, functional, dynamic and real-time bedside airway assessment with minimal radiation exposure. It helps determine a suitable size of ETT or DLT and confirm correct laryngeal mask airway (LMA) position and ETT depth [5]. It can confirm endotracheal intubation in conditions like pulmonary embolism, severe bronchospasm and cardiac arrest where capnography is unreliable or has technical problems [5,6]. It identifies airway distortions, lesions, aberrant blood vessels, trachea and cricothyroid membrane thus facilitating cricothyrotomy and tracheostomy (Figure 1). It aids the prediction of post-extubation stridor and assessment of vocal cords post thyroidectomy [7]. Two-dimensional (2-D)-USG provides sagittal and transverse plane views, three-dimensional (3-D)-USG includes the Z-axis and coronal view which permit a virtual airway reconstruction while four-dimensional USG (4-D) enables a functional airway assessment. Ultrasound predictors of a DA include non visualisation of hyoid bone, decreased hyomental distance, limited condylar mobility, increased thickness of anterior cervical soft tissue and tongue [8,9]. In view of its practicality and effectiveness, upper airway USG has potential to become established as an expected standard of care in DA management in the future.

Computed tomography (CT)

This advanced, precise modality enhances predictive accuracy of DA. Multidetector CT (MDCT) rapidly acquires high-resolution data and produces thin slice sections, 2-D and 3-D images. This enables precise localisation and finer characterisation of airway pathology like airway trauma, fractures, tumours, dislocations, oedema, foreign bodies and vocal cord palsy. Focused images, optimal visualisation, virtual views, accurate image reconstruction and determination of airway dimensions help formulate airway strategies [10].

It is especially useful in paediatric airway evaluation and diagnosis of congenital, developmental and infectious pathology as it enables quick imaging while the child is asleep or sedated without the need for airway support. In tracheomalacia, dynamic airway CT helps assess the extent of tracheal lumen collapse where a crescentic frown shape forms during expiration [10]. It is commonly used in static and dynamic airway studies, especially in OSA [11]. CT is invaluable in viewing inaccessible areas like preepiglottic and paraglottic space invasions and assessing narrowing or stenosis of airways due to laryngotracheal lesions. Preoperative CT scan helps determine the appropriate size of ETT and assess the need for a tracheostomy. For this, a three-dimensional reconstruction of the image at the level of the sixth or seventh cervical vertebral body is utilised. Using a triangulating tool, the exact point on the axial transverse section is identified in coronal and sagittal sections. Accurate measurements of the inner tracheal diameters are taken at this point in axial, coronal and sagittal planes. A spiral CT scan with multiplanar reconstruction also yields images in these planes and adjusting the angles of inclination allows these measurements to be drawn. These diameters reflect the external diameter of the ETT; a correction formula is thus applied to calculate the internal diameter of ETT. Though CT comes with its own flaws like cost and radiation exposure, it is relatively widely available and accurate. Hence, it is a commonly used invaluable resort for definitive analysis of many airway conditions and can even be used in emergencies.

Nasal Endoscopy, Virtual Laryngoscopy (VL) and VE

**Figure 2:**
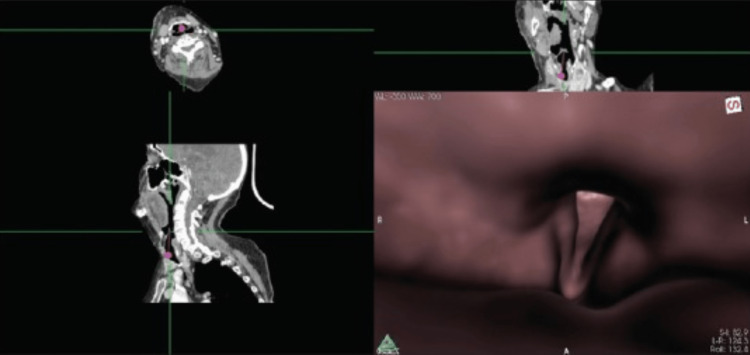
Virtual endoscopy reconstruction of glottis (bottom right) from Computed Tomography images (top and left) Source: Ahmad I, Keane O, Muldoon S. Enhancing airway assessment of patients with head and neck pathology using virtual endoscopy. Indian J Anaesth. 2017 Oct;61(10):782-786. doi: 10.4103/ija.IJA_588_17. PMID: 29242648; PMCID: PMC5664881.

Nasal endoscopy, an invasive bedside tool, facilitates visualisation of glottic and sub-glottic areas. VE and VL apply software like OsiriX and Horos to generate virtual images and 3 D airway reconstructions from CT scans (Figure 2). Accurate image acquisition, airway mapping and dynamic videos obtained improve diagnostic accuracy [12]. These non invasive modalities are invaluable for airway assessment and planning of inaccessible areas, or where airway lesions or deformities preclude airway evaluation. They can be used as an alternative where conventional methods are contraindicated. Being accurate, non-invasive and safe, this evolving modality holds great promise for assessment of challenging airway conditions.

3-D Printing

**Figure 3:**
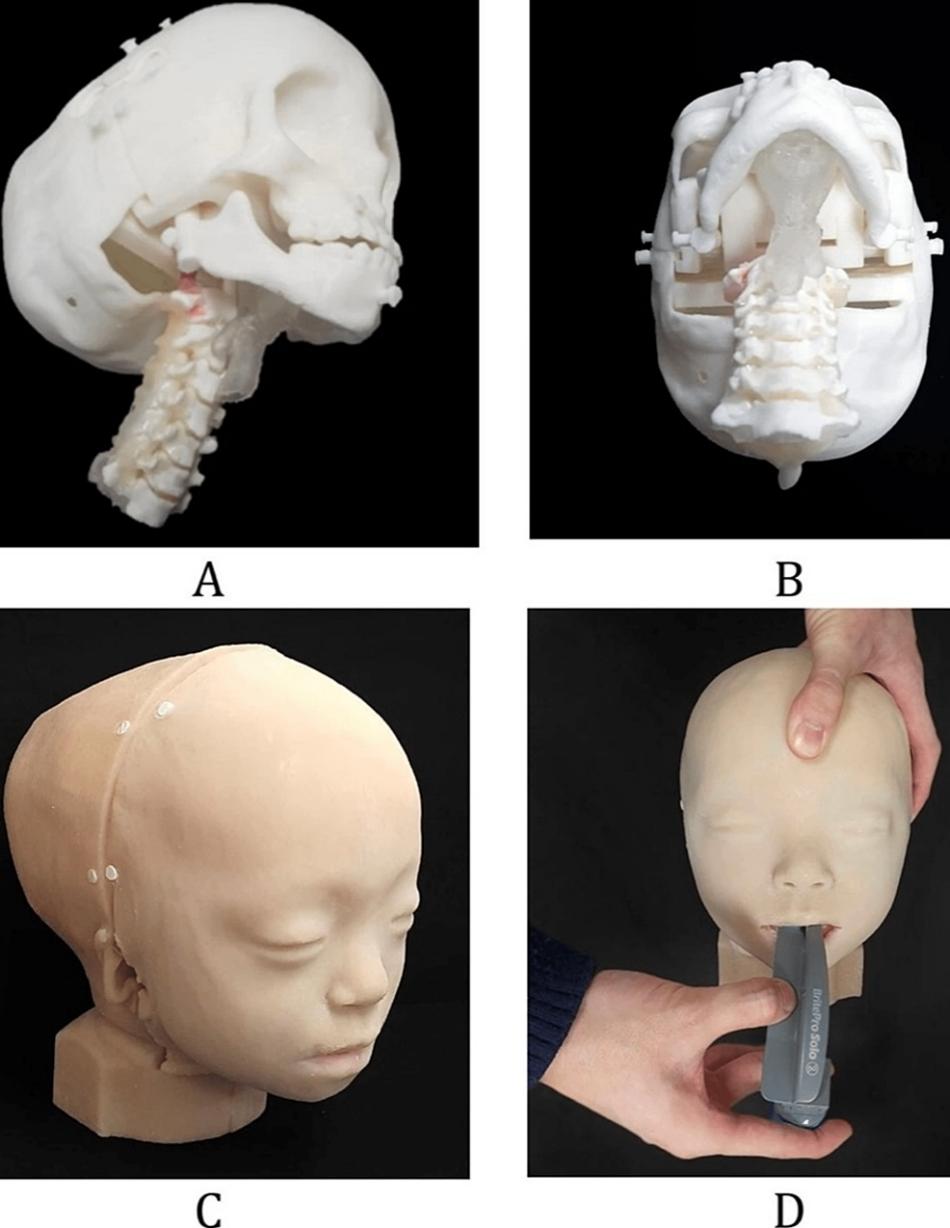
A patient-specific phantom for difficult tracheal intubation simulator. (A) and (B) Assembly of the inner parts: the skull, maxilla, mandible, cervical-spine, airway and tongue. (C) Final phantom with skin. (D) Intubation with Macintosh blades in the phantom with mouth opening. Source: Ock, J., Gwon, E., Kim, Dh. et al. Patient-specific and hyper-realistic phantom for an intubation simulator with a replaceable difficult airway of a toddler using 3D printing. Sci Rep 10, 10631 (2020). https://doi.org/10.1038/s41598-020-67575-5 This image is licensed under a Creative Commons Attribution 4.0 International License, which permits use, sharing, adaptation, distribution and reproduction in any medium or format, as long as you give appropriate credit to the original author(s) and the source, provide a link to the Creative Commons license, and indicate if changes were made. To view a copy of this license, visit http://creativecommons.org/licenses/by/4.0/.

This novel revolutionary technology utilises data from CT scans to print 3-D models of airways using 3-D rendering software like OsiriX or Pixmeo. These models are then utilised to simulate a patient’s airway; the clear anatomy provided aids airway planning of complicated cases with distorted airways due to trauma, congenital malformations or tumours (Figures 3A-3D). Critical steps can be anticipated in complex surgery of the head and neck and precise plans put in place after mutual discussions between surgeons and anaesthetists [13]. They have immense potential as tools to thoroughly study airway anatomy, practice and plan the best airway strategy and are also used in training airway skills and educating patients and families [14-16]. Wilson et al. describe the case of a paediatric one-lung ventilation where a 3-D printed model of the child’s airway was used to pre-operatively trial multiple airway devices and techniques leading to a planned and practiced successful placement of side-by-side tracheal and bronchial tubes [17]. Han et al. utilised a 3-D printed model of a post laryngectomy patient's airway to plan airway management [18]. This exciting modality is still in its infancy but has great potential in planning customised airway strategies in particularly challenging cases. It is not realistic in reproducing skin and mucosal colour changes, tissue texture, trauma, inflammation, blood or secretions. Further developments could overcome these limitations and pave the way for customised airway gadgets. Due to the low cost and practicality, it could emerge as a universal tool for airway training and patient education.

The limitations to CT and VE include secretions and blood obscuring the views. False images and measurements may be obtained based on tissue aeration and variance in tissue-air interfaces. VE and 3-D printing are time-consuming, not widely available, impractical for emergencies and hence limited to elective cases.

Magnetic Resonance Imaging (MRI)

MRI is not as useful as the other modalities for airway assessment. It helps in defining soft tissue lesions such as invasive cancers and can be used to formulate airway strategies in tumours in and around the airways [11].

Airway equipment

Existing airway equipment have undergone extensive upgrades and advanced devices have emerged (Table 2).

**Table 2:** Developments in airway devices.

Airway devices	Advantages
Direct Laryngoscopes	light emitting diode/fibreoptic light, rechargeable batteries, MRI compatible, single use versions
Video laryngoscopes	large screen, ability to record images and videos, specialised blades for difficult airways
Glidescope core	enables dual bronchoscopy and video laryngoscopy
Fibreoptic bronchoscopes	enables awake intubations, large screen, ability to record images and videos.
Optical stylets	small profile enables insertion into small mouth openings, allows for little cervical spine movement
Various versions of Supraglottic airways	ease of insertion, rescue device for ventilation, conduit for intubation, provide for the administration of volatile anaesthetics.

Direct Laryngoscopes

Numerous rigid laryngoscopy blades of alternative designs and sizes are available. Upgrades include improved illumination with light emitting diodes (LED) or fibreoptic light transmission, improved battery longevity with rechargeable options, disposable and MRI compatible versions.

Video Laryngoscopes (VLS)

**Figure 4:**
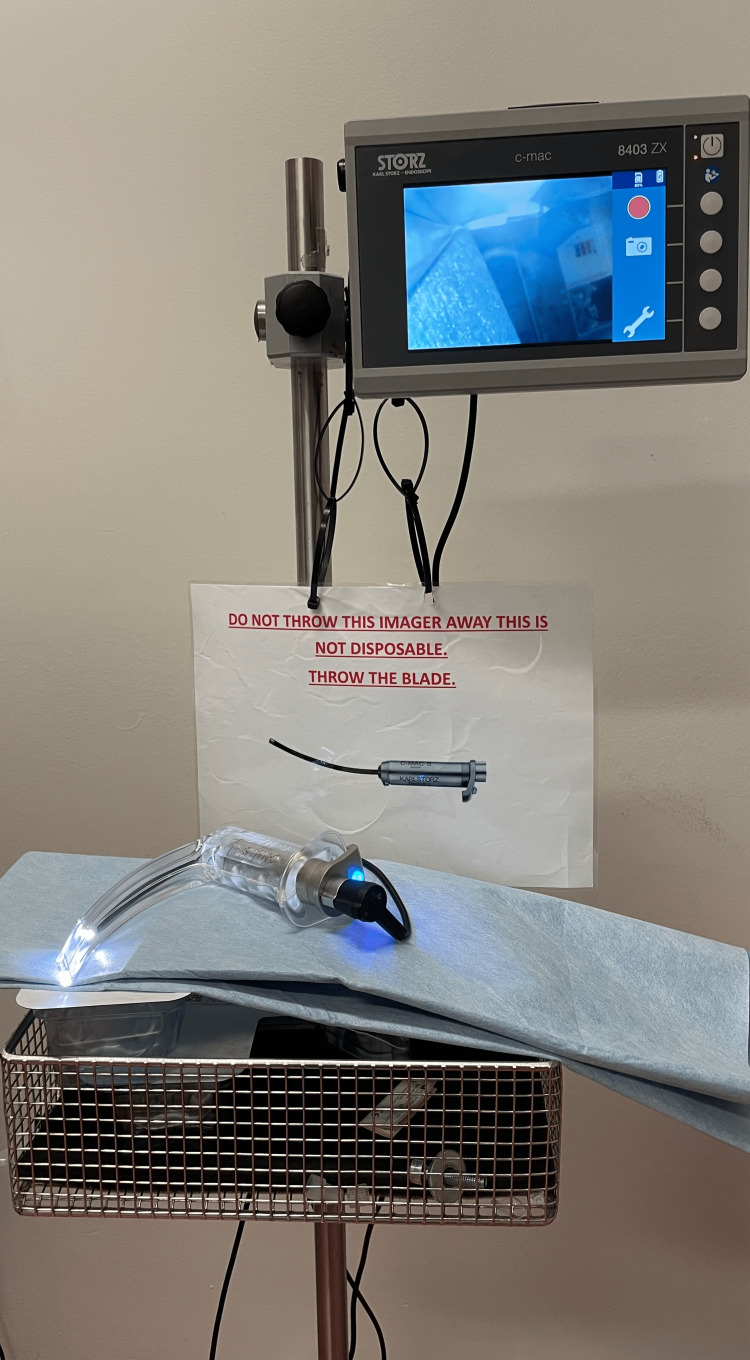
Video laryngoscope with disposable blade. The image is the original work of the author.

VLS have embedded miniature video cameras transmitting images via a fibreoptic bundle to an external monitor (Figure 4). They are popular and widely used, are battery powered and images on the monitor enable effective assistance with airway maneuvers. There are hyper angulated blades, DA blades, D blades, and X blades which provide a better view of the anteriorly placed larynx, decrease lift, require less force thus causing less haemodynamic changes. Disposable versions, antifog and image-enhancing technologies improve visualisation. Glidescope® Core™ (TECHNOPATH Distribution Ltd, Ireland) combines video laryngoscope and video bronchoscope in a single unit, provides a picture-in-picture image along with pulse oximetry and 180-degree picture rotation. Mc GRATH MAC VLS (Medtronic Limited, UK) with enhanced optics, disposable blades, durability and intelligent battery management is useful in emergencies and fast paced clinical environments. King Vision ABLADE (Ambu Ltd. UK.) and Pentax Airway Scope (Timesco Healthcare Ltd., UK) are lightweight; i-view (Intersurgical, UK) is cost effective and fully disposable with disposable batteries. Airtrac AVANT (Fannin Ltd., UK) is single use. Marshall VLS (Marshall Airway Products, UK) has ergonomic grip, full anti fog function and recordings with date and time status. CMAC (KARL STORZ SE & Co., Germany) has disposable, paediatric and DA blades and allows multiple endoscopes to be connected to one monitor. Truview (Truphatek, Israel) is a Macintosh type blade with optical lenses attached and costs similar to conventional blades. HEINE vision PRO (HEINE Optotechnik GmbH & Co. Deutschland) is durable, easy cleaning and eco-friendly. Neoscope (International Biomedical, USA) with 000, 00, 0 and 1 blades is suitable for infants under 1 kg, and facilitates non-invasive surfactant administration. MultiViewScope (MPI Medical Products International, Japan) can be used from newborn to adults, has handle, flexible scope and handle plus rigid scope for video laryngoscopy, flexible scope intubation and rigid scope intubation respectively. CMS-GS1 (Semcares, Turkey) magnifies images with preserved image quality. BD HT (Shenzhen Besdata Technology, China) has 360 degree all sight LCD, no blind spot and seven blade sizes from neonate to adult. Portable versions prove handy and useful in emergencies and remote access locations like the wards or field sites. Recording of images and videoclips aid evaluation of the procedure, planning future airway management, teaching and providing evidence in litigation claims. VLS have helped DA intubations in a variety of conditions like ankylosing spondylitis, genetic syndromes and airway masses [19-22]. VLS have truly revolutionised airway management and practically taken over as a first choice intubation aid in many countries. The future would see them integrated into airway algorithms.

Flexible Fibreoptic Bronchoscopes (FOB)

**Figure 5:**
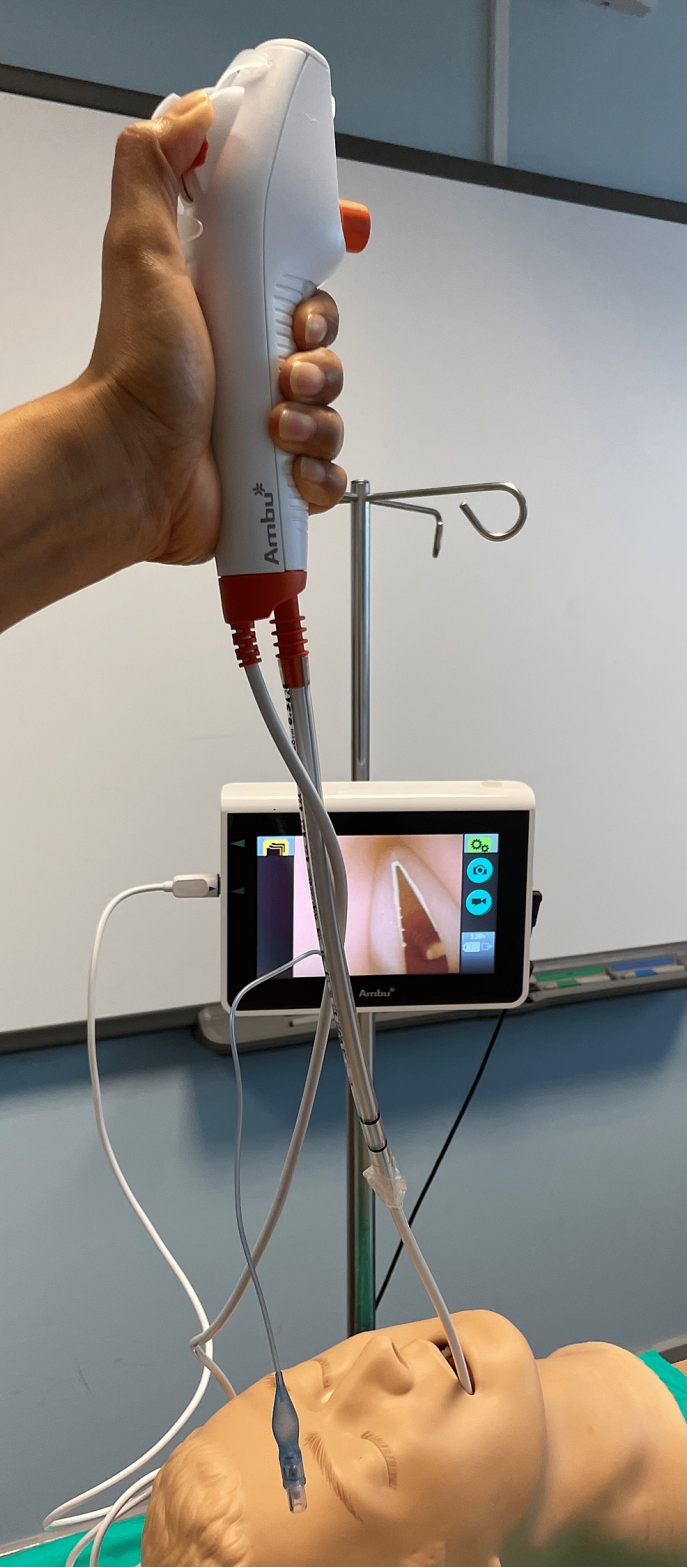
Disposable Fibreoptic Ambu aScope The image is the original work of the author.

These are versatile tools in DA management. The ASA guidelines advocate an awake intubation if the airway is deemed difficult pre-intubation, and FOB remains the most widely used technique to facilitate this. Intubations can be done nasally or orally with patients awake, sedated or asleep. An unobstructed straight view from the upper incisors to the larynx does not need to be created (Figure 5). It facilitates navigation past airways narrowed by tumours, oedema or haematoma. It is particularly useful in patients with limited mouth opening and where neck movements need to be minimised such as patients with unstable cervical spines. During a percutaneous tracheostomy, it helps ensure correct needle position, confirm correct placement and rule out false passages. There are single use versions and channels for suctioning and installing drugs or more local anaesthetic during awake intubations. FOB helps rule out and remedy mucous plugging or other causes of desaturation. The presence of blood or copious secretions cause blurring of images and precludes their use. Their use requires proper training and intubation failures can occur due to inadequate topicalisation or airway obstruction. Being invaluable diagnostic and intubation tools, these have been ingrained into anaesthetic practice.

Airway Adjuncts

These useful aids in DA management include introducers, bougies, alternative tracheal tubes, intubating stylets, and tube exchangers. Lighted or optical stylets facilitate intubation, confirmation of ETT position and tracheoscopy. The images can be viewed through an eyepiece or a video camera. They can be used independently, with laryngoscopes or with SGA. They minimise movement of the cervical spine during intubation and are useful in patients with limited oral apertures and in DA [23].

Supraglottic Airway

These lie above the glottis, provide hands-free airway maintenance and a leak-free seal for spontaneous and controlled ventilation. Patients with DA can be anaesthetised with SGA thus avoiding the potential problems associated with tracheal intubation. SGA placement cause minimal stimulation of the airway and larynx. The greater cardiovascular stability achieved on insertion and emergence benefits the physiologically compromised patient. They form Plan B of the DAS guidelines for DA management, and are advocated by the ASA DA guidelines as a rescue ventilation device.

The first-generation SGA has evolved in to newer versions and second-generation SGA. LMA with higher seal pressure and drain tube for gastric secretions, single use versions, MRI compatible SGA and flexible or reinforced SGA increase safety and prevent kinking. Intubating LMA (ILMA) allows blind tracheal intubation and fiberscope-guided intubation. The LMA CTrach (The Laryngeal Mask Company, Singapore) has integrated fibreoptic channels and a detachable viewer for viewing the larynx. It functions as an intubation conduit while minimising cervical spine movements. The laryngeal tube can be blindly inserted into the oropharynx and is useful in emergencies. An ETT can be railroaded via its ventilation port through an airway exchange catheter mounted on a fibreoptic scope. The perilaryngeal airway used as a rescue airway is designed to lodge in the hypopharynx, seals off the oropharynx and can be used as an intubation conduit. The i-gel® (Intersurgical, UK) is anatomically designed, single-use, cuffless, and is widely used in anaesthesia and resuscitation due to its ease of insertion. The unique gel-like material adapts to airway contours thus improving seal and avoiding compression trauma. It can be used as a conduit for FOB guided intubation. First-time insertion success rates are 89% and approaches 100% with three attempts [24].

Wei nasal jet tube (Well Lead Medical Equipment Ltd., China) is a simple and convenient nasal airway which can be connected directly to an anaesthesia machine. It has two inbuilt channels which maintain oxygenation and enable capnography during fibreoptic intubation and monitored anaesthesia care. It can provide active supraglottic jet ventilation during complicated DA management or “cannot intubate and cannot ventilate (CICV)” situations. The oesophageal tracheal combitube is a dual lumen airway consisting of an endotracheal tube with an oesophageal obturator allowing blind placement either in the oesophagus or the trachea. Ventilation is possible whether the device is in the oesophagus or trachea. Combination techniques of using multiple approaches or devices, for example combining direct or VLS with an optical stylet, FOB or SGA enhance the chances of successful intubation.

SGA are not definitive airways and risk airway trauma, aspiration and laryngospasm. Despite this they have been an overwhelming success having almost taken over and transformed general anaesthesia provision and saved countless lives.

Endotracheal Tube

Several novel versions and upgrades of this definitive airway have emerged. MICROCUFF Paediatric Endotracheal tubes (Avanos Medical, Inc, UK.) designed specifically for the paediatric anatomy reduce trauma and re-intubation rates. Endoflex tracheal tubes have an adjustable, bendable distal tip which permits smooth oral or nasal intubation without additional assistance or equipment. The VivaSight-DL (ETView Ltd, M P Misgav 20174, Israel) is a single-use DLT with an integrated camera in the tracheal lumen tip and a channel for clearing secretions. This allows real-time images of the airway during tracheal intubation and peri-operatively. ETT are here to stay as a device to secure the airway and further advances can be envisaged in the future.

Invasive Interventions

These include retrograde wire-guided intubation, front-of-neck percutaneous or surgical cricothyrotomy or tracheostomy, rigid bronchoscopy and extracorporeal membrane oxygenation (ECMO). These procedures are used in selected cases and are lifesaving in the event of a CICV scenario [2,25].

Retrograde intubation though infrequently used, is helpful when the upper airway is obscured by blood, tumour or obstruction and other measures have failed. A guidewire is introduced from below the glottis through the cricothyroid membrane or the membranous space between the cricoid cartilage and the first tracheal ring, directed cephalad to the mouth or nose and used to direct an ETT into the trachea. Specialised, purpose built and preassembled kits are available for retrograde intubations and cricothyroidotomy with low profile cuffs, anatomically shaped cannulas and safety clips.

**Figure 6:**
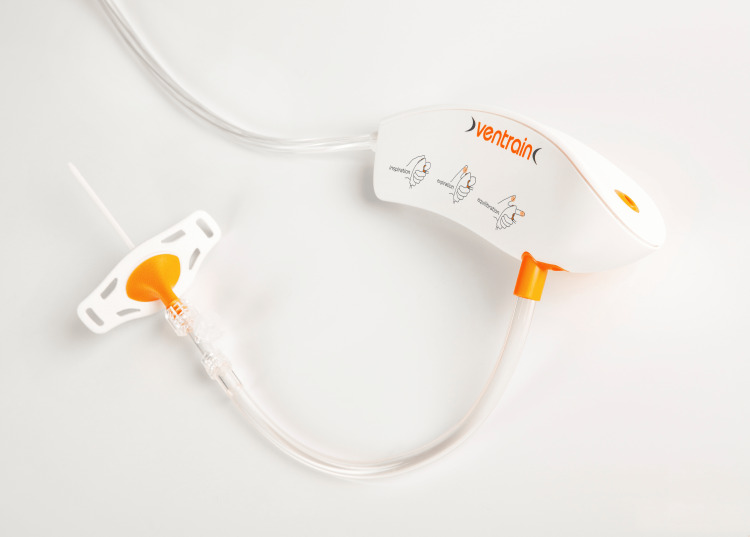
Ventrain (Ventinova, Netherlands) Courtesy Ventinova Medical B.V.

Transcutaneous transtracheal jet ventilation (TTJV) uses a catheter to insufflate oxygen or air at high pressure (0.5-4.0 bar, or 8-60 psi) into the tracheal lumen using an automatic jet ventilator or a manual trigger-activated device. The Ventrain ventilator (Ventinova Medical B.V., Netherlands), a small, hand-held, manually operated device enables ventilation via narrow-bore catheters (Figure 6). The automatic ventilator Evone (Ventinova, Netherlands) avoids the inconvenience of a hand-operated technique. It is an inspiratory flow-adjustable device generating positive pressure during inspiration and active suction during expiration through a Bernoulli effect. This avoids hyperinflation and high intrapulmonary pressures, minimises barotrauma and hemodynamic instability. These provide emergency ventilation via narrow-bore cannulas in CICV situations and during airway obstruction [26]. They maintain ventilation and oxygenation until a definitive airway is established. They improve surgical exposure and obviate the need for tracheostomy in upper airway surgery. The Tritube (Ventinova Medical B.V., Netherlands) is a narrow-bore cuffed endotracheal tube with outer diameter of 4.4 mm and a pressure measurement lumen permitting continuous intra-tracheal pressure measurements. These devices are useful in both elective and emergency situations.

Extracorporeal membrane oxygenation (ECMO) device consists of a circuit where blood is pumped out of the body to a membrane oxygenator and oxygenated blood returned to the body. It can be used electively where difficult intubation is anticipated, or as an emergency when rescue techniques are unable to sustain adequate oxygenation, and tracheostomy is not an option. It prevents the complications of a CICV situation but is time consuming to set up and needs skilled personnel, equipment and facilities.

Novel continuous oxygen supplementation techniques

Significant morbidity can occur due to hypoxemia during induction, extubation or post extubation of a DA. Advanced oxygenation techniques improve the safe apnoea time and decrease hypoxemia post extubation. High flow nasal oxygen therapy (HFNOT) or transnasal humidified rapid insufflation ventilatory exchange (THRIVE) is a method of apnoeic oxygenation and ventilation that can be used throughout the DA management including preoxygenation, induction, extubation, and post-extubation. Nasal continuous positive airway pressure mask (CPAP) is a non-invasive positive pressure oxygenation technique with a sealed nasal mask delivering high-fraction inhaled oxygen at titratable positive pressure [27,28]. These reduce anatomical dead space, provide PEEP, warmth and humidity thus improving oxygenation. They benefit a variety of settings like shared airway surgery, morbidly obese patients, bronchoscopy, sedation, etc. These have become integral in the management of at risk patients. 

Guidelines and algorithms

These have been developed worldwide by airway societies to facilitate decision-making and are based on literature analysis, surveys, research, and recommendations by experts in the field. Here we examine the guidelines from the ASA and the DAS, UK and the Vortex approach [2,25,29].

2022 American Society of Anaesthesiologists Practice Guidelines for Management of the DA

An updated DA guideline has been published by the ASA in January 2022 [3]. It was developed by representatives from several international airway societies. It stresses an intubation strategy based on familiarity, context, experience and resources available. Consideration is to be given to awake intubation and elective invasive access and use of supplemental oxygen throughout with low flow or high flow devices. The paediatric airway algorithm suggests a team-based approach with a pre-induction briefing to identify airway plans. 

DA Society 2015 Guidelines for Management of Unanticipated Difficult Intubation in Adults

A simple single algorithm has replaced the previous separate algorithms for unanticipated DA during routine intubation and during rapid sequence induction [25]. The plans proceed through plan A (intubation), B (ventilate with SGA), C (facemask ventilation/awaken) and D (invasive/front of neck access). VLS and FOB have replaced blind attempts or blind intubation through an SGA. Both guidelines stress on limiting the number of intubation attempts and recommend pre-induction briefing and debriefing at the end for feedback and reflections.

Vortex Approach

This is a simple, flexible, universal, visual cognitive aid based on the premise that facemask, ETT and SGA are the only non-surgical techniques to alveolar oxygen delivery and if best attempts at these three lifelines are unsuccessful then emergency front of neck access must be secured [29]. As recommended by all guidelines, teamwork, communication, situational awareness, flexibility and scenario-based decisions go a long way towards improving patient outcomes.

Extubation of the DA

A predetermined strategy for extubation and ensuing management of patients with a DA has been recommended by both guidelines [3,25]. Post-extubation care in Intensive care or recovery involves novel supplemental oxygenation techniques, steroids and racemic epinephrine as needed. Accurate documentation of the events, airway alert notifications and patient counselling is recommended.

Other developments

Electronic records have made access to patient’s notes from other hospitals possible. Identifying areas of difficulty and strategies which helped during previous airway management can guide future airway plans.

Artificial intelligence has made its way into the DA arena. AIMS and CDS Systems are sophisticated pieces of hardware and software technology that digitize and organize information, inform, provide solutions to and support decision making. These are hardwired into the electronic health record itself and can provide valuable feedback to clinicians in moments of importance when they are making rapid decisions. They can analyse data and detect ongoing clinical issues or deviations, and prompt alerts to help guide best practice protocols.

Advanced monitoring of beat-to-beat haemodynamics, both invasive and non-invasive, enables safer airway management of patients with disordered physiology. The revolutionary drug sugammadex with rapid and reliable reversibility of rocuronium induced paralysis is a lifesaver in DA scenarios. The drug remifentanil has simplified FOB intubation of DA by achieving deep sedation which can be rapidly reversed in case of airway compromise.

The use of explicit signage on the DA Trolley, an exact inventory, clear external labeling of drawer contents and pictorial representation of DAS algorithm eliminate mishaps to a great extent. Several versions of drawer stickers are available for use enabling rapid and easy access to equipment in a crisis scenario.

Innovations have also taken place in the way DA training is delivered. Advanced airway management fellowship programs and sub-specialities have emerged. High fidelity simulation suites, patient simulators and virtual model training provide ample opportunities to practice airway scenarios and skills. Video technology using 360-degree visuals provide a virtual learning environment and a better perception of ergonomics. Technology-enhanced learning programs like VR and augmented reality (AR) enhance the experience and provide visual feedback. VR development applications on smartphones allow the development of airway scenarios. AR goggles like HoloLens by Microsoft provide digital 3-D images, image manipulation to learn anatomy and practical experience with sensory feedback. ORSIM Bronchoscopy Simulator (ORSIM, New Zealand) is a FOB simulator with an array of DA scenarios and training modules (Video 1). It is patient and tutor free, time and cost-effective. This enables self-directed learning and hands-on practice in a safe environment. Virtual and onsite hybrid events, multi-site discussions, support networks, twitter forums, human factors training and podcast channels enhance DA learning. Online learning tools, interactive quizzes, flow charts and apps for mobile devices provide a quick reference, drug dose calculation and link to learning resources.

**Video 1:** ORSIM Bronchoscopy simulation of a difficult bronchoscopy after airway trauma (ORSIM, Airway Simulation Ltd., New Zealand) Permission granted by ORSIM, Airway Simulation Ltd., New Zealand

Toolkits and animations for patients have also been developed to enhance patient education. Conveying information to patients by video interpretation, mobile apps, telemedicine, and wearable health care technology have made the dissemination of information much more effective.

## Conclusions

Developments are evident in every aspect and stage of DA management, be it in the diagnostics, planning, airway devices, adjuncts, oxygenation techniques, guidelines and training; all designed to prevent the dreaded consequences of a poorly managed DA. The ever-expanding innovations in the anaesthetist’s armamentarium play a significant role in reducing human error, improving patient safety and aiding safer management of the DA. These leaps in technology are the need of the hour in keeping with the fact that as the complexity of cases for surgery steadily increases, so will the encounters with DA. The need to keep updated with these emerging technologies and become well-trained and skilled has consequently become a necessity to defend against litigation as well. The future will bring further technological innovations which will improve patient safety and facilitate DA management, but which also place a responsibility on the airway provider to be proficient in them.
